# Levobupivacaine Combined with Cisatracurium in Peribulbar Anaesthesia in Cats Undergoing Corneal and Lens Surgery

**DOI:** 10.3390/ani13010170

**Published:** 2023-01-01

**Authors:** Giovanna L. Costa, Fabio Leonardi, Claudia Interlandi, Filippo Spadola, Sheila Fisichella, Francesco Macrì, Bernadette Nastasi, Daniele Macrì, Vincenzo Ferrantelli, Simona Di Pietro

**Affiliations:** 1 Department of Veterinary Sciences, University of Messina, Polo Universitario Annunziata, Via Palatucci Annunziata, 98168 Messina, Italy; 2 Department of Veterinary Science, University of Parma, Via del Taglio 10, 43126 Parma, Italy; 3 Evidensia Veterinarhuset Sundsvall, Regementsvagen 9, 85238 Sundsvall, Sweden; 4 École Nationale Vétérinaire d’Alfort Unité d’anesthésie et reanimation, Pôle anesthésie et réanimation, Urgences et Soins Intensives 7, Av Du Général de Gaulle, 94700 Maisons-Alfort, France; 5 Zooprophylactic Institute, Via Gino Marinuzzi 3, 90100 Palermo, Italy

**Keywords:** peribulbar anaesthesia, levobupivacaine, cisatracurium besilate, neuromuscular blockade, corneal, lens, surgery, cats

## Abstract

**Simple Summary:**

Regional anaesthesia techniques are widely used for eye surgery and include retrobulbar and peribulbar anaesthesia; they promote adequate positioning of the eyeball. Peribulbar injection of cisatracurium and levobupivacaine provides effective akinesia and mydriasis, and shortens the onset of akinesia by prolonging its duration, without side effects and systemic neuromuscular blockade.

**Abstract:**

The aims of the study included evaluating the effects of levobupivacaine combined with cisatracurium on akinesia and mydriasis when administered by peribulbar injection, and evaluating if the chosen dose of cisatracurium is enough to avoid the use of systemic neuromuscular blockade in cats. The animals were divided into four groups as follows: group L received 1.25 mg kg^−1^ levobupivacaine administered by peribulbar injection; group LC received the same dose of levobupivacaine combined with 0.01 mg kg^−1^ of cisatracurium administered by peribulbar injection; group C received 0.01 mg kg^−1^ of cisatracurium administered by peribulbar injection; group GC received 0.01 mg kg^−1^ of cisatracurium intravenously. Physiological variables, intraocular pressure, akinesia, and mydriasis were measured before and up to 30 min after peribulbar injection. The onset of akinesia, duration of akinesia, and train of four (TOF) were evaluated. Physiological variables remained in the physiological range in all groups. Effective akinesia and mydriasis were observed in all groups. The (TOF) was 0.9 in all groups. Throughout the study was observed in group LC a shortened onset of akinesia and a prolonging its duration. The peribulbar injection of cisatracurium and levobupivacaine provided effective akinesia and mydriasis, and shortened the onset of akinesia while prolonging its duration.

## 1. Introduction

General anaesthesia usually induces ventromedial deviation of the eyeball that may hamper visualisation of the surgical field in subjects undergoing ocular surgery [1]. Nowadays, regional anaesthetic techniques, which provide akinesia, mydriasis and periocular analgesia, are commonly used for ophthalmic surgery [1,2,3,4]. Regional anaesthetic techniques include retrobulbar and peribulbar anaesthesia. Compared to retrobulbar anaesthesia, peribulbar anaesthesia reduces the incidence of haemorrhage at the level of the ocular cone and injuries to the optic nerve, and induces akinesia and mydriasis more efficiently [5,6,7,8]. The onset of akinesia after peribulbar injection of local anaesthetic drug combined with a low dose of neuromuscular blocking drug is shorter compared to that of the local anaesthetic agent alone in human beings [9,10,11,12]. Nevertheless, many serious complications (e.g., retrobulbar haemorrhage, globe perforation) may occur after retrobulbar injection. To avoid these complications, peribulbar injection has been previously used in human beings, dogs, and cats undergoing ophthalmic surgery [5,13].

Levobupivacaine is the levorotatory isomer of racemic bupivacaine. Levobupivacaine is similar to bupivacaine in duration of action and anaesthetic potency but it has fewer toxic effects on the central nervous and the cardiovascular systems [14]. Reduced toxic potential of levobupivacaine supports its use when the risk of systemic toxicity, caused by overdosing or unintentional intravascular injection, may be high (e.g., epidural or peripheral nerve blocks) [15,16]. Based on these findings, levobupivacaine has been used for peribulbar anaesthesia in elderly human patients undergoing vitreous and retinal surgical procedures [17,18,19].

Cisatracurium, an isomer of atracurium, is a non-depolarising neuromuscular blocking agent with a duration of action of approximately 27 min when administered intravenously in dogs [20,21]. Cisatracurium may be reversed by edrophonium, neostigmine, and pyridostigmine [19,20,21,22]. The clearance of cisatracurium occurs through Hoffmann elimination, bypassing hepatic metabolism and renal filtration. Consequently, cisatracurium may be safely used in patients with liver and kidney diseases.

Despite this, the use of non-depolarising neuromuscular agents and mechanical ventilators are considered safe and the standard of care for corneal and intraocular surgery in veterinary medicine [23]. However, atracurium and cisatracurium might be used in peribulbar anaesthesia to obtain extraocular muscle akinesia, as occasionally reported in the literature [9,10,11,12,23,24].

The study has the following aims: to evaluate the effects of levobupivacaine alone or combined with a low dose of cisatracurium on akinesia and mydriasis when administered via peribulbar injection; to evaluate if this dose of cisatracurium is enough to avoid the use of systemic neuromuscular blockade; to assess if this dose of cisatracurium administered by peribulbar injection may be able to cause systemic neuromuscular blockade in cats undergoing corneal and lens surgery.

## 2. Materials and Methods

The present clinical study was performed between January 2013 and December 2013. However, there was an update of bibliographic references consulted for this article. The study was approved by the Review Board for Animals Care of the University of Messina, which provided consent to the clinical study with consent/assent: Italian Law (Circular 26/02/2007 Administrative procedures relating to the conduct of clinical investigations with CE marked medical devices). Procedures were performed in accordance with Italian Law (D.M. 116192), European Law (O.J. of E.C. L 358/1 12/18/1986), and USA Laws (Animal Welfare Assurance No A5594-01, Department of Health and Human Services, Arlington, VA, USA). The owners signed a voluntary informed consent form prior to the cats’ enrolment in the study [20].

Thirty female and ten male cats, aged 3 ± 2 years, and weighing 3.5 ± 0.5 kg were enrolled in the study. The inclusion criteria were patients who were to undergo corneal (penetrating corneal ulcers, *n* = 32) and lens surgery(cataract, *n* = 8), with normal packed cell volume and biochemical parameters. All cats were sedated using butorphanol 0.2 mg kg^−1^ (Dolorex 1%; Intervet, Aprilia, Italy) combined with dexmedetomidine 15 mg kg^−1^ (Dexdomitor 0.5%, Pfizer Animal Health, Rome, Italy) and midazolam 0.2 mg kg^−1^ (Ipnovel 0.5% Roche, Basel, Switzerland) administered intramuscularly. After approximately 15 min, a 0.64 × 19 mm, 24G venous catheter (DELTA VEN, Deltamed, Viadana, Italy) was inserted in the cephalic vein for medication and fluid administration. Artificial tear eye drops were applied on corneal surface every thirty minutes, until the start of surgery (Artelac splash; Baush & Lomb, Marcherio, Italy). Anaesthesia was induced with 2 mg kg^−1^ of propofol (Proposure 1%, Merial, Assago, Italy) intravenously (IV) over 30–60 s. All cats received 2 mg kg^−1^ of lidocaine (Lidocaina 2%, Zoetis, Rome, Italy) sprayed on the glottis and endotracheal intubation was performed with a cuffed tube. Anaesthesia was maintained with isoflurane (Isoflo, Esteve, Barcelona, Spain) delivered in 100% oxygen via a rebreathing circle system. Ventilation was supported by intermittent positive pressure ventilation. The mechanical ventilator (Servoventilator 900 C, Siemens Elema, Sweden) was set using the following parameters: respiratory rate 20 breaths min^−1^_,_ tidal volume 16/18 mL kg^−1^, inspiratory/expiratory ratio (I:E) 1:2, and airway pressure 20 cmH_2_O.

Then, the patients were randomly assigned to one of four treatment groups (*n* = 10) by drawing a ticket. All cats were administered peribulbar anaesthesia using a 0.4 × 13 mm 27G sterile needle (Latex Free Benefis, Genoa, Italy). The anaesthetic mixture was applied both in the inferior temporal corner and in the upper nasal corner by inserting the needle between orbit and eyeball [6]. The group L received levobupivacaine 1.25 mg kg^−1^ (0.75% Chirocaina, Abbott, Chicago, IL, USA); the group LC received 1.25 mg Kg^−1^ of levobupivacaine combined with cisatracurium 0.01 mg kg^−1^ (0.2% Nimbex, Glaxo SmithKline, Verona, Italy); the group C received 0.01 mg kg^−1^ of cisatracurium; the group GC received 1 mL of saline solution (S.A.L.F. spa Laboratorio Farmacologico, Bergamo, Italy) between orbit and eyeball and cisatracurium IV (0.01 mg kg^−1^). The drugs used were diluted in normal saline at the same volume of 1 mL/eye [25]. All peribulbar anaesthesia was performed using the same volume of drug (1 mL), with the addition of saline solution 0.9%. If ineffective eyeball block occurred or any discomfort was detected (the cut off point for rescue analgesia was the increase of 20% of the physiological parameters), additional local anaesthetic peribulbar block with levobupivacaine was administered. A single observer blinded to treatment recorded the following parameters: heart rate (HR—beats min^−1^), end-tidal carbon dioxide tension (EtCO_2—_mmHg), arterial haemoglobin oxygen saturation (SpO_2_—%), non-invasive oscillometric blood pressure (NIBP, systolic, mean, diastolic—mmHg) by placing a blood pressure cuff, approximately 30/40% of the circumference of the tail, around the base of the tail, and the concentration of inspired and expired isoflurane (IT/ET isoflurane—%) using a multiparameter monitor (AMI s.r.l., Leonardo model, Milan, Italy).

The evaluation of neuromuscular transmission was performed by detecting the TOF (train of four) using a machine (TOF-Watch^®^ SX, Organon, Italy) automatically set before each use at 50 mA and 1/0.1 Hz. Subcutaneous stimulating electrodes were applied at the medial part of the elbow (at the level of the ulnar nerve), whereas subcutaneous recording electrodes were applied above the carpus (near the accessory carpal bone).

All parameters were measured before sedation (time 0), at 15 min after sedation (S) (except TOF, SpO_2_, and EtCO_2_), and at 5, 10, 15, 20, 25, and 30 min after peribulbar anaesthesia.

Intraocular pressure (IOP—mmHg) was measured using a tonometer (Tono-Pen Vet, Reichert, Italy). IOP baseline values were recorded after instillation of one drop of oxybuprocaine (0.4% Novesina, Novartis, Italy) in awake animals. Afterward, IOP was measured at 15 min after sedation (S), and at 5 and 10 min after peribulbar anaesthesia with cats in sternal recumbency. The degree of mydriasis was evaluated by measuring the horizontal pupil diameter (mm) using a Jameson calliper (E2410, Storz^®^, Italy) in awake animals, at 15 min after sedation (S), and at 5 and 10 min after peribulbar anaesthesia. The horizontal pupil diameter was measured in the same room lit with a 40W lamp. Eyeball centralisation and akinesia were evaluated by three observers blinded to treatment, considering the eye as rotated if any deviation from central position was noted. Centralisation of the eye denoted akinesia of the globe and eyelids.

All cats were supposed to be hospitalised in case of any complications during recovery.

Statistical analysis was performed using SPSS 15.0 IBM software for Windows. Kendall’s test of concordance and the Shapiro–Wilk test were performed. The data, expressed as median and range, were compared using the Friedman test to evaluate changes along the time line and to compare differences between all groups. Statistical significance was set at *p* < 0.05. Power calculation of sample was performed using Sample Size Calculator software: confidence level 5%; population proportion 50%.

## 3. Results

Data were not normally distributed. The sample is representative of the cat population operated on at our hospital within 12 months, undergoing intraophthalmic surgery. No anaesthetic complications were encountered.

High level of concordance inter-observer (W = 1) was recorded in all groups, and data were not normally distributed. The sample of subjects enrolled in the present study is not enough representative of the population. HR, NIBP, EtCO_2_, and SpO_2_ remained within physiological ranges throughout surgery and no significant differences were recorded between groups. HR ranged between 95 and 130 bpm, systolic NIBP between 110 and 130 mmHg, mean NIBP between 80 and 97 mmHg, diastolic NIBP between 75 and 95 mmHg, EtCO_2_ between 32 and 34 mmHg, and SpO_2_ between 98 and 100%. The concentration of inspired and expired isoflurane ranged between 0.5 and 1% in all groups.

Table 1 showed ophthalmic variables recorded in the groups. The onset of akinesia in group LC was significantly shorter compared to group L (*p* = 0.000).

The duration of akinesia in group LC was significantly longer compared to those of the other groups (*p* = 0.000). The duration of akinesia in group L was significantly longer compared to those of groups C and GC (*p* = 0.000). The duration of akinesia in group C was significantly longer compared to group GC (*p* = 0.000). In groups C and GC, additional peribulbar injection of levobupivacaine was required.

IOP significantly decreased in all groups. IOP was significantly lower in group LC compared to group L at 5 and 10 min after peribulbar anaesthesia (*p* = 0.000). In groups GC and C, IOP significantly decreased after sedation but no significant differences were recorded after peribulbar or intravenous cisatracurium administration.

The horizontal pupil diameter was significantly higher in group LC compared to group L at 5 and 10 min after peribulbar anaesthesia (*p* = 0.000). The horizontal pupil diameter significantly increased after sedation in group GC. The horizontal pupil diameter significantly increased in group C after peribulbar cisatracurium administration (*p* = 0.000). The horizontal pupil diameter was significantly higher in group C compared to group GC (*p* = 0.000). Throughout the study, the TOF (T1:T4) was 0.9 in all groups.

## 4. Discussion

The present study aimed to evaluate the synergism between a local anaesthetic agent (levobupivacaine) and a neuromuscular blocking agent (cisatracurium), and to rule out the potential systemic effect of a low dose of cisatracurium.

In the present study, levobupivacaine combined with cisatracurium and administered by peribulbar injection appeared to provide a clinically effective degree of mydriasis, rapid eyeball centralization, and akinesia, as well as maintenance of normal IOP values during the surgery [9,10,13]. Even though only corneal and lens surgeries were performed, it is likely that eyeball centralisation, akinesia, and mydriasis combined with a normal IOP, which is mandatory for the success of ophthalmic surgery, may facilitate also the phacoemulsification and intraocular procedures. The onset and duration of eyeball centralisation and akinesia of levobuvicaine combined with cisatracurium were faster and longer compared to those of levobupivacaine alone [11,12,23]. It is likely that these effects may be due to the presence of the Felderstruktur, an anatomical ocular structure, provided with small grape-like nerve endings, which responds with a slow tonic contraction to non-depolarising agents, such as cisatracurium [11,12,23,26].

As expected, the administration of a low dose of neuromuscular blocking agent combined with a local anaesthetic drug in peribulbar injection allowed for use of low doses of isoflurane without systemic neuromuscular blockade [23,26]. Peribulbar anaesthesia is a reliable alternative to retrobulbar anaesthesia [5,6,7]. Few side effects were previously recorded with either anaesthetic technique [6,7], but compared retrobulbar anaesthesia, peribulbar anaesthesia reduces the incidence of haemorrhage at the level of the ocular cone and injuries to the optic nerve [5,6,7]. As regard with analgesia and akinesia, no clear differences between peribulbar and retrobulbar anaesthesia were demonstrated in human beings. Shilo-Benjamini et al. (2013) highlighted that single peribulbar technique was better than double peribulbar and retrobulbar techniques in cats, because single peribulbar injection provided “large” distribution of injected drug and, consequently, it could determine 86% of regional anaesthesia. The increase in IOP has been previously described after peribulbar injection, but the result was obtained in a cadaveric study, so the in vivo changes in IOP may be different [5]. On the contrary, in the present study, IOP significantly decreased after peribulbar injection. The likely reason of this result is the different sample population. Nevertheless, it may be also due to the lower injected volume compared to that previously used [5]. Even though a regional anaesthetic technique was used, general anaesthesia was required. Consequently, also the anaesthetic management may have influenced the recorded variables. As previously reported, dexmedetomidine combined with midazolam and butorphanol reduced IOP and induced mydriasis [27,28]. The postsynaptic activation of α-adrenoceptors in the central nervous system due to dexmedetomidine and the changes in sympathetic activity due to butorphanol may have decreased basal IOP. Moreover, it cannot be ruled out that the combination of dexmedetomidine and butorphanol induced mild hypotension that was responsible for decreased in IOP [29,30,31,32].

As previously reported, no systemic neuromuscular blockade was recorded in all groups by measuring TOF. However, if neuromuscular blocking agents are administered, the use of an automatic ventilator is advisable.

The present study has some limitations. Firstly, the number of cases recruited for cataract surgery was lower compared to the number of cats undergoing corneal surgery. Secondly, the present study is a clinical and non-experimental study and, consequently, many clinical variables (e.g., aetiology, previous medical treatment, time between the appearance of lesion and time of surgery) may have influenced the results. Unfortunately, it is very difficult to recruit a number of subjects with similar surgical lesions. Thirdly, meaningful references dealing with the effects of low dose of neuromuscular blocking agents combined with local anestahetic drugs administered by peribulbar injection are lacking in the veterinary literature.

## 5. Conclusions

Peribulbar anaesthesia combined with low doses of isoflurane is an effective method for anesthetising cats undergoing corneal and lens surgeries. The addition of 0.01 mg kg^−1^ of cisatracurium to peribulbar administration of levobupivacaine improves the onset and duration of the local block and the degree of mydriasis but it reduces IOP. Cisatracurium administered through peribulbar injection does not cause systemic neuromuscular blockade. In future studies, post-peribulbar anaesthesia IOP could be evaluated using a sedation protocol that does not interfere with this.

## Figures and Tables

**Table 1:** Ophthalmic variables recorded in the groups: Legend: IOP, intraocular pressure; LC, levobupivacaine 2.5 mg kg^−1^ combined with cisatracurium 0.01 mg kg^−1^ (peribulbar); L, levobupivacaine 2.5 mg kg^−1^ (peribulbar); GC, cisatracurium 0.01 mg kg^−1^ (EV); C, cisatracurium 0.01 mg kg^−1^ (peribulbar); 0′, before sedation; S, 15 min after sedation; 5′–30′, measurements after peribulbar anaesthesia and cisatracurium IV administration; underlined, significant differences between values at different times, *p* < 0.05; bold, significant differences between groups, *p* < 0.05.

Measured Data expressed as median (range) IOP (mmHg) Horizontal pupil diameter (mm) Akinesia and centralization.	*Groups* *LC* *L* *GC* *C* *LC* *L* *GC* *C* *LC* *L* *GC* *C*	*0′* ***20(16/22)*** ***18(16/20)*** ***17(16/18)*** ***18(16/20)*** *5(4/6)* *5(4/6)* *4(4/6)* *6(4/6)*	*S* ***18(16/20)*** ***17(15/19)*** ***16(16/17)*** ***18(16/20)*** *6(5/7)* *6(5/7)* ***5(5/6)*** *6(5/7)* Eye rotated Eye rotated Eye rotated Eye rotated	*5′* ***16(15/17)*** ***17(15/19)*** ***16(15/17)*** ***18(17/18)*** ***8(7/9)*** ***6(5/7)*** ***5(5/6)*** ***8(7/9)*** Akinesia Eye rotated Akinesia Akinesia	*10′* ***15(13/17)*** **17(15/19)** ***16(15/17)*** ***16(15/17)*** ***8(7/9)*** **6(5/7)** ***5(5/6)*** ***8(7/9)*** Akinesia Akinesia Akinesia Akinesia	*15′* Akinesia Akinesia Akinesia Akinesia	*20′* Akinesia Akinesia Akinesia Akinesia	*25′* Akinesia Akinesia Akinesia Akinesia	*30′* Akinesia Akinesia Akinesia Akinesia
Onset of akinesia (min.) Duration of akinesia (min.)	*LC* *L* *GC* *C* *LC* *L* *GC* *C*	***4(3/5)*** ***8(6/10)*** *5(5/5)* *5(3/7)* ***70(68/72)*** ***60(/57/63)*** ***5(5/6)*** ***20(20/20)***							

## Data Availability

The data presented in this study are available on request from the corresponding author.
